# Differential activation of spinal and parabrachial glial cells in a neuropathic pain model

**DOI:** 10.3389/fncel.2023.1163171

**Published:** 2023-04-04

**Authors:** Valeria Mussetto, Aurora Moen, Lidia Trofimova, Jürgen Sandkühler, Roni Hogri

**Affiliations:** Department of Neurophysiology, Center for Brain Research, Medical University of Vienna, Vienna, Austria

**Keywords:** neuropathic pain, neuroinflammation, microglia, astrocytes, lateral parabrachial nucleus, spinal cord, chronic constriction injury (CCI)

## Abstract

The clinical burden faced by chronic pain patients is compounded by affective comorbidities, such as depression and anxiety disorders. Emerging evidence suggests that reactive glial cells in the spinal cord dorsal horn play a key role in the chronification of pain, while supraspinal glia are important for psychological aspects of chronic pain. The lateral parabrachial nucleus (LPBN) in the brainstem is a key node in the ascending pain system, and is crucial for the emotional dimension of pain. Yet, whether astrocytes and microglia in the LPBN are activated during chronic pain is unknown. Here, we evaluated the occurrence of glial activation in the LPBN of male Sprague–Dawley rats 1, 4, and 7 weeks after inducing a chronic constriction injury (CCI) of the sciatic nerve, a prevalent neuropathic pain model. CCI animals developed mechanical and thermal hypersensitivity that persisted for at least 4 weeks, and was mostly reversed after 7 weeks. Using immunohistochemical staining and confocal imaging, we found that CCI caused a strong increase in the expression of the astrocytic marker GFAP and the microglial marker Iba1 in the ipsilateral spinal dorsal horn, with peak expression observed 1 week post-injury. Moreover, morphology analysis revealed changes in microglial phenotype, indicative of microglia activation. In contrast, CCI did not induce any detectable changes in either astrocytes or microglia in the LPBN, at any time point. Thus, our results indicate that while neuropathic pain induces a robust glial reaction in the spinal dorsal horn, it fails to activate glial cells in the LPBN.

## 1. Introduction

Chronic pain affects up to one third of the population, and is associated with enormous societal and economic costs (Institute of Medicine, 2011; Loggia et al., 2015). In addition to the sensory component of pain, chronic pain patients often suffer from complex affective comorbidities, such as anxiety disorders, sleep disturbances, and depression (Radat et al., 2013). The emotional components of pain drastically reduce these patients’ quality of life and complicate their clinical course and treatment responsiveness (Leo, 2005). Despite being subject to intense investigation, the neurobiological mechanisms underlying chronic pain affective disturbances are still poorly understood. In recent years, a large body of clinical and preclinical evidence has indicated that the activation of glial cells, in particular astrocytes and microglia, represents a common substrate of both chronic pain and its psychological comorbidities, constituting a potential mechanistic link (Walker et al., 2014; Xanthos and Sandkühler, 2014).

In the context of chronic pain, glial activation has been extensively studied in the spinal cord dorsal horn (Grace et al., 2014). Activated astrocytes and microglia, and an increase in pro-inflammatory mediators, have been observed in the dorsal horn following peripheral nerve injury, and contribute to the development of pain hypersensitivity (Ji et al., 2013). However, given that the neural circuits mediating the cognitive and affective components of pain are supraspinal, it is at this level that glia would be expected to contribute to the emergence of pain affective comorbidities (Fiore and Austin, 2016). Indeed, in addition to their effects on spinal glial cells, peripheral neuropathies drive aberrant spinal output, potentially leading to the release of pro-inflammatory substances that impact both glial cells and neurons in downstream targets (Sandkühler, 2009; Xanthos and Sandkühler, 2014). Recent evidence indicates that neuropathic insults induce changes in cytokine or chemokine expression and alter glial cell activity in brain sites that likely encode the emotional aspects of chronic pain (Norman et al., 2010; Ren et al., 2011; Di Cesare Mannelli et al., 2013; Dellarole et al., 2014; Nascimento et al., 2015; Di Cesare et al., 2017; Barcelon et al., 2019). Different patterns of regional neuro-glial-immune interactions could therefore differentially contribute to various aspects of pain (Yalcin et al., 2014; Liu et al., 2017; Sideris-Lampretsas and Malcangio, 2021).

One of the major relay stations between nociceptive dorsal horn neurons and higher brain regions is the lateral parabrachial nucleus in the brainstem (LPBN). Moreover, the LPBN is widely recognized as a hub that integrates sensory and motivational information to modulate pain and emotional behavior (Palmiter, 2018; Chiang et al., 2019). Dysregulated neuronal activity in the LPBN has been observed in neuropathic pain model animals (Uddin et al., 2018; Raver et al., 2020; Sun et al., 2020), suggesting that neuronal adaptations in the LPBN might underlie some aspects of pain pathology. However, contrary to other brain regions involved in pain processing, little is known about how chronic pain affects LPBN glia. In the current study, we examined astrocytic and microglial activation in the LPBN of rats following a peripheral nerve injury. Given that emotional disturbances of chronic pain usually develop at later stages following an injury (Yalcin et al., 2011; Sieberg et al., 2018; Barcelon et al., 2019), we examined pain behavior and glial activation both at early and delayed time points after nerve injury. We report that CCI induces long-lasting mechanical and thermal hypersensitivity, as well as glial activation in the dorsal horn, but not in the LPBN.

## 2. Methods

### 2.1. Animals

All experiments were performed on male Sprague-Dawley rats aged 40–45 days at the beginning of the habituation phase. Animals were bred in-house at the Medical University of Vienna, or purchased from Janvier Laboratories (Saint-Berthevin Cedex, France). Animals were kept under standard conditions, with unrestricted access to food and water and a 12 h light/dark cycle. Experiments were performed during the light phase. All procedures were performed in accordance with the European Communities Council directives (86/609/EEC) and approved by the Austrian Federal Ministry of Education, Science and Research (BMBWF).

### 2.2. Chronic constriction injury surgery

Chronic constriction injury surgery was performed as previously described (Bennett and Xie, 1988; Draxler et al., 2021). Briefly, rats were deeply anaesthetized with isoflurane and the left sciatic nerve was exposed, proximal to the trifurcation. Three non-resorbable ligatures were tied ∼1 mm apart around the nerve using chromic gut (CP medical, Norcross, GA, USA). Muscle and skin were sutured (silkam 6/0 and prolene 4/0, Ethicon) and clipped. In sham surgeries, the sciatic nerve was similarly exposed but no ligation was made.

### 2.3. LPS challenge

As a systemic inflammatory challenge, 5 mg/kg of lipopolysaccharide (LPS; from *E. coli* 0111:B4, Sigma) dissolved in saline solution was injected i.p. Control animals received i.p. injections of saline solution. Animals were killed 24 h following injection. We selected the dose and time course of the LPS challenge based on previous literature (Hoogland et al., 2015) and on our own pilot experiments.

### 2.4. Behavioral testing

Hindpaw withdrawal responses to mechanical and heat stimulation were measured in multiple daily sessions using the von Frey and Hargreaves tests, respectively, as previously described (Draxler et al., 2021). Following a 2-day habituation, two baseline sessions were performed on two consecutive days. One day later (day 0), CCI or sham surgery was performed. Next, test sessions were repeated as stated in the text and figures. Experimenters were blinded to the treatment received by each rat.

#### 2.4.1. Von Frey test

Rats were placed in acrylic boxes on a wire mesh floor. Calibrated von Frey filaments (Stoelting, Wood Dale, IL, USA) of varying force were applied to the plantar surface of the left and right hindpaw (5 stimulations per paw) and used to assess the 50% paw withdrawal threshold using the simplified up and down method (Bonin et al., 2014).

#### 2.4.2. Hargreaves test

Rats were placed in acrylic boxes on a glass surface. Heat sensitivity was assessed by application of a radiant heat beam (100–130 mW/cm^2^) to the plantar surface of each hindpaw (5 min inter-trial interval, 3 stimulations per paw) *via* the Hargreaves apparatus (Stoelting, Wood Dale, IL, USA), for a maximum of 20 s per trial. The mean withdrawal latency was calculated.

### 2.5. Immunohistochemistry

Twenty-four hours following the last testing day, rats were killed with sodium-pentobarbital (100 mg/kg, i.p) and transcardially perfused with 1% heparinized ice-cold saline solution, followed by 4% paraformaldehyde (PFA, 7.4 pH). Tissue was extracted and stored in PFA at 4°C overnight, and then cryoprotected in 20 and 30% sucrose. Coronal slices (40 μm thick) of either L4–L5 segments of the spinal cord or the brainstem containing the LPBN were cryosectioned (CM3050S, Leica, Germany) and stored in 0.05% Na-azide phosphate-buffered saline (PBS) solution for free-floating immunohistochemical labeling. Slices were treated with 0.1% PBS-Triton X-100 and blocked with 4% normal donkey serum (Abcam, UK) for 1 h. Slices were incubated overnight at room temperature with the following primary antibodies: rabbit anti-glial fibrillary acidic protein (anti-GFAP; 1:500; Cell Signaling) and goat anti-ionized calcium-binding adapter molecule 1 (anti-Iba1; 1:500, Abcam). Next, slices were incubated with the following secondary antibodies for 2 h: donkey anti-rabbit conjugated to Alexa Fluor 546 (1:500, ThermoFisher, Waltham, MA, USA) and donkey anti-goat conjugated to Alexa Fluor 647 (1:500, ThermoFisher). Finally, slices were mounted onto microscope slides using a mounting medium containing 49, 69-Diamidin-2-phenylindol (DAPI; ThermoFischer), and cover-slipped.

### 2.6. Confocal microscopy and image analysis

Images were obtained with an inverted confocal microscope (Leica TCS SP5) using a 20× glycerol immersion objective. Z stacks (15 μm and 1 μm step) were acquired at a resolution of 1024 × 1024 pixels using excitation laser lines 405, 561, and 633 nm. All settings were kept identical within each experiment. For each animal, 3–4 sections of the L4–L5 lumbar spinal cord and 3–4 sections spaced along the rostro-caudal axis of the LPBN were processed. To image the spinal dorsal horn, one picture per side was taken for each slice; to image the LPBN, two pictures per side were taken for each slice and stitched together using the pairwise stitching of ImageJ plugin (Preibisch et al., 2009). All image analysis was performed offline using Fiji ImageJ (version: 1.53) by experimenters blinded to the treatment received by each rat. For each image, a maximum intensity projection was generated and a region of interest (ROI) was drawn either around laminae I-III of the dorsal horn or around the LPBN. GFAP and Iba1 signals were binarized using a fixed threshold, and the percentage of pixels above the threshold within the ROI was measured to assess immunofluorescence density (% Area fraction). Iba1+ cells were counted manually for each slice. In addition, microglial morphology was analyzed using skeleton analysis as previously described (Morrison and Filosa, 2013; Hadschieff et al., 2020), to measure the number of branches and the average branch length per microglial cell. For a step by step protocol, please refer to Young and Morrison (2018). Briefly, binarized images were converted into skeletonized images and each positive pixel in the skeleton was categorized as an end point, slab, or juncture pixel, based on the number of positive neighboring pixels (1, 2, and >2, respectively) using the AnalyzeSkeleton ImageJ plugin. Adjacent slab pixels were counted to assess the length of branches in μm. For each skeleton item in the ROI, the number of pixels of each type, as well as the mean branch length, was determined. To exclude small cellular fragments, items with ≤2 endpoints and a branch length shorter than 1 μm were not included in the analysis. Next, the total number of endpoints and branch length within the ROI was normalized by Iba1+ cell count as a measure of the number of extremities per cell and of the length of microglial branches, respectively.

### 2.7. Data and statistical analysis

All statistical analyses were performed and plotted using GraphPad Prism 9 (GraphPad Software, San Diego, CA, USA). Group sizes were calculated taking into account the type and number of planned comparisons, as well as the expected variability, based on previous experience and pilot data. Hindpaw withdrawal thresholds and latencies were measured on multiple days. Baseline values were calculated as the average of 2 days of testing prior to treatment. A two-way mixed design ANOVA was performed, with treatment (CCI or sham surgery) and time (relative to surgery) as independent variables. When appropriate, *post hoc* analyses were used to perform the following comparisons: (1) baseline vs. each post-treatment testing day; (2) baseline threshold values between treatments; (3) threshold values during the last testing day between treatments. To analyze immunohistochemical data from CCI- and sham-treated rats, a two-way mixed design ANOVA was performed for each time point (1, 4, and 7 weeks), using treatment and side (ipsilateral or contralateral to surgery) as independent variables. When appropriate, *post hoc* analyses were used to perform: (1) a within-group comparison between the two sides; (2) a between-group comparison for each side. All *post hoc* comparisons were performed using the Holm-Sidak multiple comparisons test. For immunohistochemistry following LPS experiments, data from the left and right side were averaged for each animal and LPS- and vehicle-treated rats were compared with independent samples *t*-tests. Data are presented as mean ± standard error of the mean (SEM), and *p*-values < 0.05 were considered statistically significant. *P*-values in the main text and figures refer to *post hoc* comparisons, or *t*-test results. The results of all statistical tests are summarized in Supplementary Table 1 (behavior) and Supplementary Table 2 (immunohistochemistry).

## 3. Results

### 3.1. CCI induces long-lasting mechanical and thermal hypersensitivity in the injured hindlimb

**FIGURE 1:**
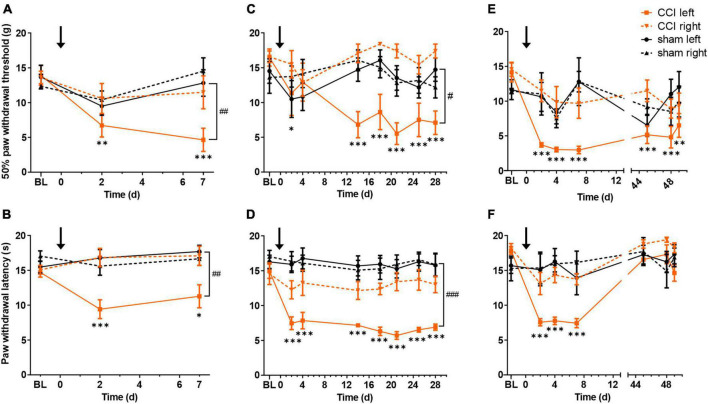
Chronic constriction injury (CCI) of the left sciatic nerve induces long lasting mechanical and heat hypersensitivity. Mechanical paw withdrawal thresholds and radiant heat withdrawal latencies are shown for 1 week (**A,B**, respectively), 4 weeks **(C,D)** or 7 weeks **(E,F)** following CCI (orange) or sham (black) surgery. Full lines indicate left paw (ipsilateral to surgery), dashed lines right paw (contralateral to surgery). BL indicates mean value of two baseline days. Black arrow indicates time of surgery (day 0). *p < 0.05, ^**^p < 0.01, and ^***^p < 0.001, when comparing each testing day to baseline, for the CCI-treated hindlimb; ^#^*p* < 0.05, ^##^*p* < 0.01, and ^###^*p* < 0.001, when comparing CCI- and sham-treated hindlimbs on the last testing day. *n* = 6 rats per group.

Compared to controls, mechanical and thermal hypersensitivity were observed in the injured hind limbs of CCI rats at 1 week (Figures 1A, B) and 4 weeks (Figures 1C, D). CCI-induced reduction of mechanical paw withdrawal thresholds as compared to baseline persisted up to 7 weeks post-surgery; however, no significant difference between CCI and sham-treated rats could be detected at this late time point (*p* = 0.07; Figure 1E). CCI-induced thermal hypersensitivity was completely reversed at 7 weeks post-surgery (Figure 1F). No difference was found in baseline values between treatments. In summary, CCI surgery induced localized pain hypersensitivity that lasted for at least 4 weeks, and partially recovered after 7 weeks.

### 3.2. CCI induces strong gliosis in the ipsilateral spinal dorsal horn

**FIGURE 2:**
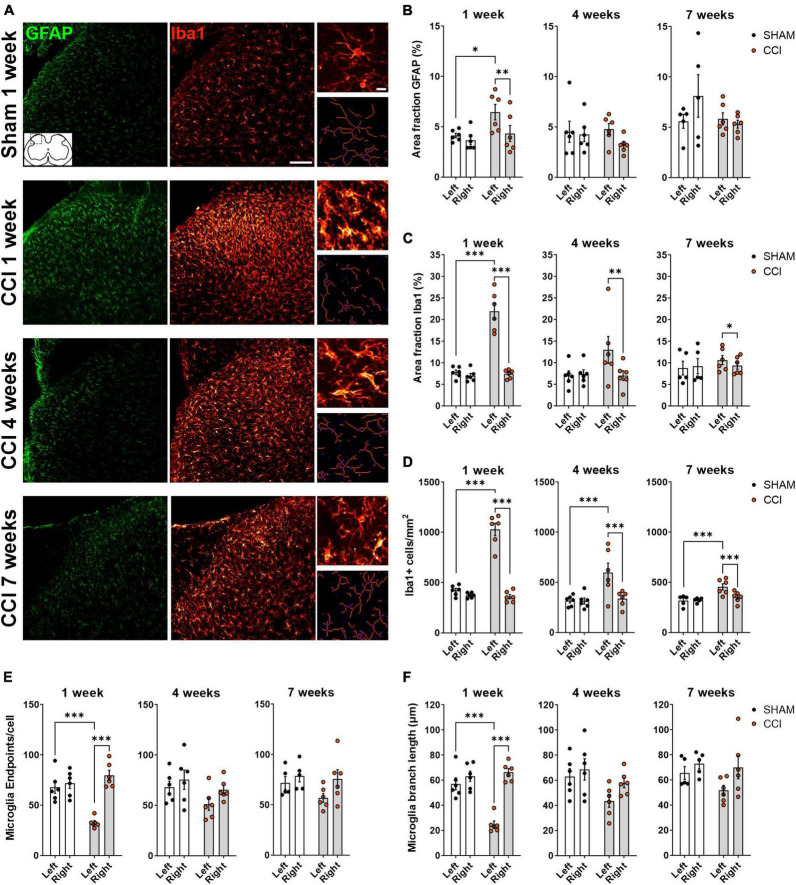
CCI induces glial activation in ipsilateral spinal dorsal horn that partially subside over time. **(A)** Representative images of the left spinal dorsal horn, ipsilateral to CCI or sham surgery, showing immunohistochemical labeling of the astrocytic marker GFAP (green) and the microglial marker Iba1 (red). Insets on the right show high-magnification images of microglial cells (top), and the corresponding skeletons (bottom) used for quantitative evaluation of microglia morphology. Scale bars: 100 μm for overview images, 10 μm for insets. **(B,C)** Immunofluorescence density (Area fraction) of GFAP **(B)** and Iba1 **(C)** at 1, 4 and 7 weeks following surgery. **(D–F)** Quantification of microglial cell number per mm^2^ **(D)** and microglial morphology **(E,F)** revealed a strong microgliosis and a deramified phenotype of microglia, as indicated by a reduction in the number of extremities [endpoints, **(E)**] and in branch length **(F)**. Microglial activation peaked 1 week following CCI induction, and elevated microglia density was still detected at 7 weeks. *p < 0.05, ^**^p < 0.01, and ^***^p < 0.001, *n* = 6 rats per treatment, 3–4 slices per animal.

We next sought to examine whether neuropathic pain behavior is associated with glial activation in the spinal dorsal horn, and whether glial activation is restricted to the side ipsilateral to the injury. Astrocytic and microglial activation were examined at three different time points (1, 4, or 7 weeks post-surgery), by staining for GFAP and Iba1, respectively (Figure 2A). One week post-surgery, GFAP density, measured as % area fraction, was increased in the ipsilateral but not contralateral dorsal horn of CCI rats (CCI ipsi vs. CCI contra: *p* = 0.004; CCI ipsi vs. sham ipsi: *p* = 0.026). No increase in GFAP area fraction was observed 4 or 7 weeks post-surgery (Figure 2B). Iba1 area fraction reached its peak 1 week after CCI induction (*p* < 0.0001 for CCI ipsi as compared to both CCI contra and sham ipsi). Four and seven weeks post-surgery, Iba1 area fraction was still significantly increased in the ipsilateral spinal cord of CCI rats as compared to the contralateral side (4 weeks: *p* < 0.007; 7 weeks: *p* = 0.018), but was not significantly different from the ipsilateral spinal cord of sham-operated rats (4 weeks: *p* = 0.063; 7 weeks: *p* = 0.56; Figure 2C).

Microglial activation is associated with cell proliferation and changes in microglial phenotype as they switch from a ramified resting state to an amoeboid-deramified state. As proliferation and deramification might have opposing effects on iba1 immunofluorescence, we also measured microglial cell density and evaluated microglia morphology using skeleton analysis (Morrison and Filosa, 2013; Young and Morrison, 2018). At all time points tested (1, 4, and 7 weeks post-surgery), microglia density was significantly elevated in the ipsilateral dorsal horn of CCI-treated rats, both compared to the contralateral dorsal horn and to the ipsilateral dorsal horn of sham-operated rats (Figure 2D). CCI treatment induced a significant reduction of endpoints/cell, indicating a reduced number of microglia extremities, in the ipsilateral dorsal horn 1 week post-surgery, but not at later time points (Figure 2E). Similarly, a strong reduction in microglial branch length was observed in the ipsilateral spinal dorsal horn of CCI rats at 1 week, but not 4 or 7 weeks post-surgery (Figure 2F). Taken together, these results indicate that CCI induces a unilateral activation of both astrocytes and microglia in the dorsal horn, lasting for at least 1 week, with microglia cell density remaining elevated for at least 7 weeks.

### 3.3. CCI failed to induce any detectable changes in LPBN glial cells

**FIGURE 3:**
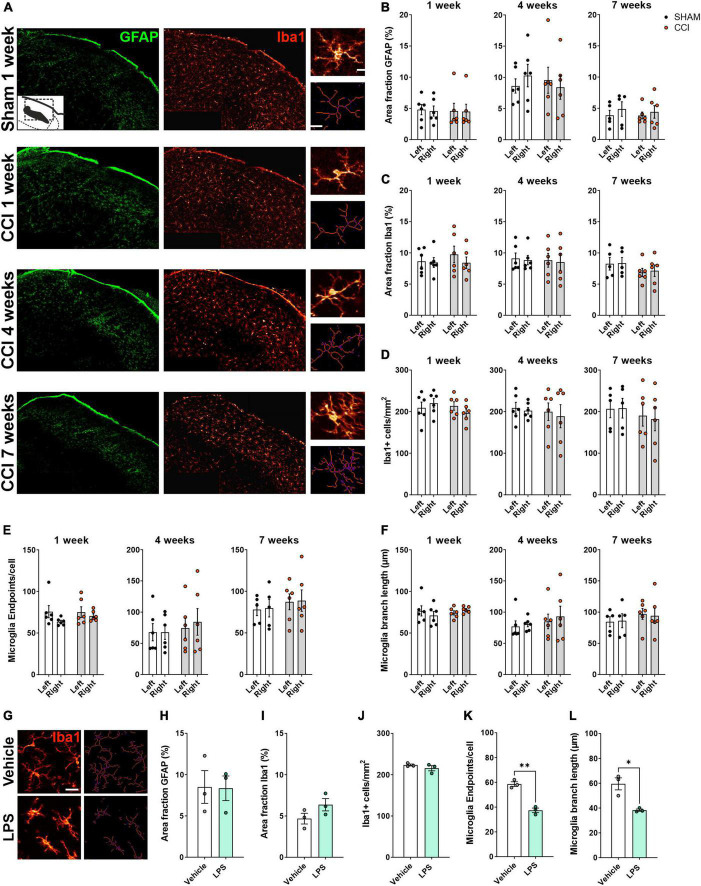
Glial activation was not detected in the LPBN of rats following CCI surgery. **(A)** Representative images of the right LPBN, showing immunohistochemical staining for the astrocytic marker GFAP (green) and the microglial marker Iba1 (red). Insets on the right show high-magnification images of microglial cells (top), and the corresponding skeletons (bottom) used for quantitative evaluation of microglia morphology. Scale bars: 100 μm for overview images, 10 μm for inset. **(B,C)** Immunofluorescence density (Area fraction) of GFAP **(B)** and Iba1 **(C)** at 1, 4, and 7 weeks following surgery. **(D–F)** Quantification of microglial cell number per mm^2^ **(D)** and microglial morphology **(E,F)** revealed no changes at 1, 4, or 7 weeks following surgery. *n* = 6 rats per treatment, 3–4 slices per animal. **(G–N)** A systemic inflammatory challenge induces detectable microglial activation in the LPBN. **(G)** Representative images of Iba1+ cells (left) and the corresponding skeletons (right) following i.p. administration of LPS (5 mg/kg) or vehicle. Scale bar: 25 μm. LPS did not affect GFAP **(H)** or Iba1 **(I)** immunofluorescence density and microglial cell density **(J)**, but significantly reduced the number of extremities [endpoints, **(K)**] and the average branch length **(L)** in microglia cells. *p < 0.05 and ^**^p < 0.01, *n* = 3 rats per treatment, 3–4 slices per animal, average of left and right LPBN.

Next, we asked whether CCI-induced glial activation could also be detected in the LPBN (Figure 3A). GFAP and Iba1 area fraction were not significantly different in the CCI group as compared to the sham group at any time point tested (Figures 3B, C), in neither the ipsilateral nor the contralateral LPBN. Similarly, CCI did not induce any significant change in microglia cell density (Figure 3D), number of endpoints per cell (Figure 3E), or branch length (Figure 3F). Thus, the glial activation we observed in the spinal dorsal horn was not reflected by similar changes in LPBN glial cells.

To confirm that our method is sensitive enough to detect glial cell activation in the LPBN, we systemically administered LPS, or saline as a vehicle control, in a separate cohort of rats. We chose LPS to induce a physiological form of systemic inflammation. LPS produces robust and pervasive inflammation, including microglial and astrocytic activation within the CNS (Hoogland et al., 2015; Perez-Dominguez et al., 2019; Diaz-Castro et al., 2021). In particular, LPS was previously shown to cause the secretion of pro-inflammatory mediators and neuronal activation within the LPBN (Tkacs and Li, 1999; Zhang et al., 2003; Cheng et al., 2022). While LPS administration failed to induce any change in GFAP and Iba1 area fraction, or in microglial cell number (Figures 3G–J), a significant reduction of endpoints/cell (Figure 3K) and branch length (Figure 3L) was found in the LPBN of rats receiving LPS compared to those receiving vehicle, pointing to a deramified, activated phenotype of microglial cells in the LPBN following a systemic inflammatory challenge.

Collectively, these data suggest that while parabrachial microglial cells can be activated during certain conditions, glia are not activated by the CCI neuropathic pain model in the rat.

## 4. Discussion

The LPBN is a key region for the processing of noxious and aversive sensory inputs (Palmiter, 2018; Chiang et al., 2019). Importantly, neuropathic pain is correlated with increased activity in parabrachial neurons (Uddin et al., 2018; Deng et al., 2020; Raver et al., 2020). This suggests that long-lasting adaptations in this area contribute to the hypersensitivity and enhanced aversion associated with chronic pain. Glial cells have been implicated in several forms of plasticity at key regions involved in nociception and pain (Ji et al., 2013; Fiore and Austin, 2016), and we have recently described a form of opioid-induced plasticity in the LPBN that is dependent on glial cells (Mussetto et al., 2023, Under review). However, the involvement of LPBN glia in neuropathic pain has not been previously addressed. In the current study, we show that chronic constriction injury of the sciatic nerve, a well-known model for neuropathic pain, induces strong and long lasting gliosis in the spinal dorsal horn, but fails to activate glial cells in the LPBN.

### 4.1. Spinal glial activation

In the spinal dorsal horn, reactive astrocytes and microglia have been consistently found to participate in the induction and maintenance of chronic pain (Ji et al., 2013). Increased expression of spinal GFAP and Iba1 has been observed in several pain models, including neuropathic pain models (Wen et al., 2011; Ji et al., 2013, 2018; Inoue and Tsuda, 2018). Consistent with previous reports, we observed that CCI induces an intense upregulation of both markers, as well as microglial proliferation and deramification, in the ipsilateral dorsal horn. Previous reports have shown that microglial activation usually occurs within a few days following peripheral nerve injury and gradually subsides (Inoue and Tsuda, 2018), while astrocytic activation occurs later and lasts for months (Di Cesare et al., 2014; Li et al., 2020; Kurabe et al., 2022), suggesting that microglia are involved in the induction of chronic pain, while astrocytes might have a more important role in its maintenance (Raghavendra et al., 2003; Milligan and Watkins, 2009; Chen et al., 2014). In contrast, under our experimental conditions, we observed that both microglial and astrocytic reaction peaked 1 week after surgery, and while some evidence for microglial activation was still observed after 7 weeks, GFAP upregulation was completely reversed at both later time points. Such differences could be related to the nature and duration of the aberrant peripheral input (Xanthos and Sandkühler, 2014). Indeed, in the current study pain hypersensitivity was mostly resolved 7 weeks post-CCI; therefore, the lack of sustained astrocytic reactivity at later time points might reflect a process of recovery.

### 4.2. Supraspinal glial activation

In addition to glial activation at the spinal dorsal horn, peripheral insults can affect glial cell activity, as well as cytokine and chemokine expression, in brain regions that likely encode the emotional aspects of chronic pain (Ren et al., 2011; Dellarole et al., 2014; Yalcin et al., 2014; Nascimento et al., 2015; Barcelon et al., 2019). Interestingly, glial activation is observed in specific and discrete brain regions, and can differentially affect neurons in different areas (Liu et al., 2017; Taylor et al., 2017; Barcelon et al., 2019; Sideris-Lampretsas and Malcangio, 2021). In various animal models of chronic pain, astrocytic activation has been observed in the ACC (Ikeda et al., 2013; Yamashita et al., 2014), primary somatosensory cortex (Ishikawa et al., 2018), periaqueductal gray [PAG; (Mor et al., 2010, 2011; Norman et al., 2010; Dubový et al., 2018)], rostral ventromedial medulla (Wei et al., 2008; Roberts et al., 2009; Dubový et al., 2018), and amygdala (Marcello et al., 2013). However, the latter study reported no changes in astrocytic GFAP expression across several other regions, including the prefrontal cortex and the PAG (Marcello et al., 2013). Reports on brain microglia activation in chronic pain are also variable. Zhang et al. (2008) found no evidence of microglia activation in several brain region following spared nerve injury. In contrast, a recent study observed differential activation of microglia following CCI surgery in several forebrain regions, including the hippocampus, the amygdala and the prefrontal cortex, but no changes in brainstem regions such as the PAG (Barcelon et al., 2019). However, microglial activation in the PAG has been detected in neuropathic rats in other studies (Takeda et al., 2009; Chu et al., 2012). One possible reason for such discordant findings might be the timing of tissue extraction. Presumably, injury-induced aberrant activity would gradually spread along the neuraxis, such that neurogenic inflammation and glial activation might occur in the brain at later stages compared to the spinal cord (Xanthos and Sandkühler, 2014; Barcelon et al., 2019), and the exact timeline of glial activation might substantially differ between brain regions.

Here, we provide the first report examining the effects of CCI on LPBN astrocytes and microglia, at both early and later time points following neuropathic injury. Although we can’t exclude the possibility that the time points used in this study missed a narrow time window of LPBN glial activation, this interpretation seems unlikely. In particular, when analyzing microglia, we utilized multiple parameters to assess both proliferation and morphological changes. With regard to astrocytes, GFAP is commonly used as an activation marker due to its robust upregulation observed across several pathological conditions, including peripheral nerve injuries (Hol and Pekny, 2015). Nonetheless, use of additional activation markers, especially for astrocytes, or different techniques such as gene expression profiling or *in vivo* calcium imaging might detect more subtle changes in glial activity following neuropathic injury.

The results of our LPS experiment demonstrate that LPBN microglia can indeed react to pro-inflammatory mediators. Interestingly, inflammatory mediators such as interleukin-6 (Mishra et al., 2019) and prostaglandin-2 (Skibicka et al., 2011; Cheng et al., 2022), presumably of glial origin (Mishra et al., 2019), have been shown to modulate behavior and neuronal activity at the parabrachial level (Skibicka et al., 2011; Mishra et al., 2019; Cheng et al., 2022). It remains to be elucidated whether individual differences in glial responses exist in the LPBN. Recently, Fiore and Austin (2018) have shown glial adaptations in the hippocampus and prefrontal cortex that were restricted to a subgroup of rats that developed long-lasting emotional disturbances following nerve injury, suggesting that individual susceptibility to glial adaptations might underlie similar individual vulnerability to chronic pain-induced affective symptoms. Additional work would be required to evaluate whether priming parabrachial glial cells, for example *via* LPS, might increase the responsiveness of LPBN glia to neuropathic injury and worsen the emotional impact of chronic pain. This might give mechanistic insight into why individuals with increased inflammatory reactivity might be more prone to developing chronic pain and related affective disturbances (Walker et al., 2014; Xanthos and Sandkühler, 2014; Fiore and Austin, 2016).

### 4.3. Limitations

The present study was performed on male rats. Of note, glial response in nerve injury-induced neuropathic pain is sexually dimorphic (Mogil, 2020). Spinal cord microglia, which is critical in the induction of chronic pain in males, may not be relevant for pain hypersensitivity in females (Sorge et al., 2015). In contrast, astrocytic involvement appears to be sex-independent (Chen et al., 2018). Additionally, neuropathic pain of different etiologies, such as chemotherapeutic or diabetic, might differentially affect brain glial cells (Di Cesare et al., 2017; Dubový et al., 2018; Lee and Kim, 2020; Lu et al., 2021). Thus, additional studies would be required to assess the generalizability of the present findings.

## 5. Conclusion

Expanding on previous findings, the present study indicates that a peripheral neuropathy can drive region-specific glial activation, suggesting a differential contribution of glia to specific aspects of pathological pain. Interestingly, glial activation was found in specific and discrete pain-related brain regions in human chronic pain patients (Loggia et al., 2015; Albrecht et al., 2021). Therefore, a deeper understanding of regional variations in glial responses might help identify new markers to guide clinical strategies.

## Data availability statement

The raw data supporting the conclusions of this article will be made available by the authors, without undue reservation.

## Ethics statement

The animal study was reviewed and approved by the Committee for Animal Experimentation of the Medical University of Vienna, and by the Austrian Federal Ministry of Education, Science and Research (BMBWF).

## Author contributions

VM, JS, and RH conceived the project. VM, AM, and LT performed the research. VM and RH wrote the manuscript. All authors commented on the final version of the manuscript and approved its submission.
