# Supplementary material for: Differential activation of spinal and parabrachial glial cells in a neuropathic pain model
Source: Front Cell Neurosci. 2023 Apr 4;17:1163171. doi: 10.3389/fncel.2023.1163171 (PMC10110840; doi:10.3389/fncel.2023.1163171)
Supplement: Supplementary file 1 [file Data_Sheet_1.PDF]

Supplementary Material

Differential activation of spinal and parabrachial glial cells in a neuropathic pain model

Valeria Mussetto[1], Aurora Moen[1], Lidia Trofimova[1], Jürgen Sandkühler[1], Roni Hogri[1*]

[1] Department of Neurophysiology, Center for Brain Research, Medical University of Vienna, Vienna, Austria

*** Correspondence:**
Roni Hogri
roni.hogri@meduniwien.ac.at

**Behaviour**			**ANOVA**			**Post hoc comparisons (p)**							
						Within treatment (Baseline vs last day)				Between treatment (Baseline)		Between treatment (Last day)	
			Time	Treatment	Time x Treatment interaction	CCI left	CCI right	sham left	sham right	CCI left vs Sham left	CCI left vs CCI right	CCI left vs Sham left	CCI left vs CCI right
**Von Frey**	1 week	F	F (2, 40) = 7.5	F (3, 20) = 2.4	F (6, 40) = 2.7								
		p	**0.0017**	0.093	**0.027**	**0.0002**	0.5364	0.9055	0.5239	0.9995	0.9992	**0.0037**	**0.0186**
	4 weeks	F	F (7, 140) = 3.6	F (3, 20) = 3.8	F (21, 140) = 4.1								
		p	**0.0014**	**0.027**	**<0.0001**	**<0.0001**	0.9988	>0.9999	0.9721	0.8374	>0.999	**0.0109**	**0.0003**
	7 weeks	F	F (6, 108) = 4.7	F (3, 18) = 11.5	F (18, 108) = 1.6								
		p	**0.0003**	**0.0002**	0.065	**0.0069**	0.1313	>0.9999	0.9917	0.6778	0.9963	0.0732	0.5391
**Hargreaves**	1 week	F	F (2, 38) = 1.33	F (3, 19) = 8.29	F (6, 38) = 3.289								
		p	0.277	**0.001**	**0.0105**	**0.0289**	0.2682	0.2014	0.9578	0.9521	0.9945	**0.0005**	**0.0016**
	4 weeks	F	F (7, 140) = 5.7	F (3, 20) = 18.7	F (21, 140) = 2.3								
		p	**<0.0001**	**<0.0001**	**0.0021**	**<0.0001**	0.8300	0.9998	0.9584	0.8213	0.9955	**<0.0001**	**0.0014**
	7 weeks	F	F (6, 108) = 11.1	F (3, 18) = 8.5	F (18, 108) = 3.1								
		p	**<0.0001**	**0.001**	**0.0001**	0.3404	>0.9999	0.949	0.9563	0.5901	0.9958	0.407	0.1398

Table S1 Statistical results of behavioral data. Data was analyzed using a two-way mixed design ANOVA, followed by the Holm-Sidak post-hoc tests.

IHC spinal cord		ANOVA						Post hoc comparisons			
		Side		Treatment		Side x treatment interaction		between sides (left vs right)		between treatments (CCI vs Sham)	
								CCI	sham	left	right
		F	p	F	p	F	p	p	p	p	p
GFAP area fraction	1 week	F (1, 10) = 12.63	**0.0052**	F (1, 10) = 3.78	0.080	F (1, 10) = 5.01	**0.049**	**0.0043**	0.61	**0.0264**	0.68
	4 weeks	F (1, 10) = 6.021	**0.034**	F (1, 10) = 0.18	0.68	F (1, 10) = 3.02	0.11	-	-	-	-
	7 weeks	F (1, 9) = 1.218	0.30	F (1, 9) = 1.075	0.33	F (1, 9) = 2.86	0.13	-	-	-	-
Iba1 area fraction	1 week	F (1, 10) = 64.33	**<0.0001**	F (1, 10) = 44.87	**<0.0001**	F (1, 10) = 51.55	**<0.0001**	**<0.0001**	0.57	**<0.0001**	0.75
	4 weeks	F (1, 10) = 6.61	**0.03**	F (1, 10) = 1.45	0.26	F (1, 10) = 8.20	**0.017**	**0.0065**	0.97	0.0625	0.99
	7 weeks	F (1, 9) = 2.17	0.17	F (1, 9) = 0.29	0.60	F (1, 9) = 8.90	**0.015**	**0.0182**	0.56	0.56	1.00
Iba1+ cell	1 week	F (1, 10) = 111.6	**<0.0001**	F (1, 10) = 63.95	**<0.0001**	F (1, 10) = 83.25	**<0.0001**	**<0.0001**	0.55	**<0.0001**	0.96
	4 weeks	F (1, 10) = 13.21	**0.005**	F (1, 10) = 5.40	**0.043**	F (1, 10) = 12.64	**0.005**	**0.001**	1.00	**0.0025**	0.94
	7 weeks	F (1, 9) = 7.55	**0.023**	F (1, 9) = 8.45	**0.017**	F (1, 9) = 9.09	**0.015**	**0.0041**	0.98	**0.0015**	0.61
endpoints/cell	1 week	F (1, 10) = 82.37	**<0.0001**	F (1, 10) = 5.001	**0.0493**	F (1, 10) = 58.56	**<0.0001**	**<0.0001**	0.56	**<0.0001**	0.46
	4 weeks	F (1, 10) = 11.40	**0.007**	F (1, 10) = 2.22	0.17	F (1, 10) = 1.08	0.32	-	-	-	-
	7 weeks	F (1, 9) = 7.36	**0.024**	F (1, 9) = 1.12	0.32	F (1, 9) = 1.57	0.24	-	-	-	-
branch length/cell	1 week	F (1, 10) = 106.3	**<0.0001**	F (1, 10) = 9.34	**0.012**	F (1, 10) = 59.14	**<0.0001**	**<0.0001**	0.18	**<0.0001**	0.79
	4 weeks	F (1, 10) = 7.61	**0.020**	F (1, 10) = 3.67	0.085	F (1, 10) = 1.47	0.25	-	-	-	-
	7 weeks	F (1, 9) = 8.69	**0.016**	F (1, 9) = 1.20	0.30	F (1, 9) = 1.54	0.25	-	-	-	-

IHC LPBN		Side		Treatment		Side x treatment interaction		between sides (left vs right)		between treatments (CCI vs Sham)	
								CCI	sham	left	right
		F	p	F	p	F	p	p	p	p	p
GFAP area fraction	1 week	F (1, 10) = 0.067	0.80	F (1, 10) = 0.013	0.91	F (1, 10) = 0.014	0.91	-	-	-	-
	4 weeks	F (1, 10) = 0.17	0.69	F (1, 10) = 0.038	0.85	F (1, 10) = 5.04	**0.049**	0.23	0.17	0.71	0.71
	7 weeks	F (1, 9) = 3.24	0.11	F (1, 9) = 0.042	0.84	F (1, 9) = 0.23	0.64	-	-	-	-
Iba1 area fraction	1 week	F (1, 10) = 2.43	0.15	F (1, 10) = 0.16	0.69	F (1, 10) = 1.37	0.27	-	-	-	-
	4 weeks	F (1, 10) = 0.99	0.34	F (1, 10) = 0.046	0.84	F (1, 10) = 0.002	0.97	-	-	-	-
	7 weeks	F (1, 9) = 0.54	0.48	F (1, 9) = 1.15	0.31	F (1, 9) = 0.14	0.71	-	-	-	-
Iba1+ cell	1 week	F (1, 10) = 0.14	0.71	F (1, 10) = 0.43	0.53	F (1, 10) = 3.56	0.09	-	-	-	-
	4 weeks	F (1, 10) = 1.02	0.34	F (1, 10) = 0.19	0.67	F (1, 10) = 0.066	0.80	-	-	-	-
	7 weeks	F (1, 9) = 0.34	0.57	F (1, 9) = 0.36	0.56	F (1, 9) = 0.72	0.42	-	-	-	-
endpoints/cell	1 week	F (1, 10) = 3.64	0.09	F (1, 10) = 0.31	0.59	F (1, 10) = 0.71	0.42	-	-	-	-
	4 weeks	F (1, 10) = 1.24	0.29	F (1, 10) = 0.28	0.61	F (1, 10) = 1.28	0.28	-	-	-	-
	7 weeks	F (1, 9) = 0.16	0.69	F (1, 9) = 0.40	0.54	F (1, 9) = 0.002	0.97	-	-	-	-
branch length/cell	1 week	F (1, 10) = 0.20	0.66	F (1, 10) = 0.17	0.69	F (1, 10) = 5.14	**0.047**	0.23	0.16	0.70	0.45
	4 weeks	F (1, 10) = 1.86	0.20	F (1, 10) = 0.44	0.52	F (1, 10) = 0.26	0.62			-	-
	7 weeks	F (1, 9) = 0.023	0.88	F (1, 9) = 0.53	0.48	F (1, 9) = 0.27	0.62			-	-

i.p LPS IHC LPBN	t-test	
	p	t, df
GFAP area fraction	0.953	t=0.063, df=4
Iba1 area fraction	0.163	t=1.705, df=4
Iba1+ cell	0.313	t=1.153, df=4
endpoints/cell	**0.0012**	t=8.147, df=4
branch length/cell	**0.0125**	t=4.316, df=4

Table S2 Statistical results of histological data. Data from CCI and sham animals was analyzed using a two-way mixed design ANOVA, followed by the Holm-Sidak post-hoc test when appropriate. Data from LPS- and vehicle-injected animals was analyzed using an unpaired t-test.
